# Sex-Related Differences in Heart Failure Development in Patients After First Myocardial Infarction: The Role of Galectin-3

**DOI:** 10.3390/biomedicines12122661

**Published:** 2024-11-21

**Authors:** Milica Dekleva, Tamara Djuric, Ana Djordjevic, Ivan Soldatovic, Aleksandra Stankovic, Jelena Suzic Lazic, Maja Zivkovic

**Affiliations:** 1 Faculty of Medicine, University of Belgrade, 11000 Belgrade, Serbia; dekleva.milica@gmail.com (M.D.); soldatovic.ivan@gmail.com (I.S.); jsuzic@yahoo.com (J.S.L.); 2 Laboratory for Radiobiology and Molecular Genetics, VINČA Institute of Nuclear Sciences—National Institute of the Republic of Serbia, University of Belgrade, 11000 Belgrade, Serbia; tamariska@vin.bg.ac.rs (T.D.); alexas@vin.bg.ac.rs (A.S.); majaz@vin.bg.ac.rs (M.Z.); 3 Cardiology Department, Clinic for Internal Medicine, University Clinical Hospital Center “Dr. Dragisa Misovic-Dedinje”, 11000 Belgrade, Serbia

**Keywords:** heart failure, myocardial infarction, galectin-3, *LGALS-3* mRNA, gal-3 ELISA

## Abstract

**Backgrounds:** Galectin-3 (gal-3) is upregulated in remodeling, and failing myocardium and gal-3 levels are increased in hypertrophy, fibrosis and inflammation. The aim of this study was to investigate the potential role of sex-related differences in the following: risk factors, left ventricular (LV) structural and functional changes, coronary angiography, expression of the gal-3 encoding gene *LGALS-3* and plasma gal-3 levels in heart failure (HF). **Materials and Methods**: This prospective study included 137 men and 44 women with first MI who underwent Doppler echocardiography within 2–4 days of MI and after 6 months. Relative *LGALS-3* mRNA expression in peripheral blood mononuclear cells (PBMCs) was detected using TaqMan^®^ technology. Plasma gal-3 concentration was determined by ELISA method. **Results**: In the acute phase of MI, LV end-diastolic and end-systolic volume indexes (LVEDVI and LVESVI) were significantly lower in women compared to men (58.2 ± 13.1 vs. 46.3 ± 11.1, *p* < 0.001; 33.7 ± 9.5 vs. 27.0 ± 9.2, *p* < 0.001, respectively). The incidence of LV hypertrophy (LVH) and HF was significantly higher in women compared to men (70.0% vs. 44.6%, *p* = 0.03; 37.5% vs.19.5%, *p* = 0.02, respectively). There was a significant correlation between the grade of LV diastolic dysfunction (LVDD) and plasma gal-3 levels (*p* < 0.001). The relative expression of *LGALS-3* mRNA in PBMCs was higher in females (fold induction = 1.326, S.E. range = 0.748–2.587, *p* = 0.007). Plasma gal-3 levels were higher in women compared to men (44.66 ± 28.04 vs. 16.30 ± 12.68, *p* < 0.001) and higher in patients with HF than in patients without HF (31.14 ± 27.09 vs.21.39 ± 18.17, *p* = 0.025). **Conclusions**: Gender-specific factors such as LVH, LVDD, *LGALS-3* mRNA expression and plasma gal-3 levels may explain the increased incidence of HF in women. The differences in the model and determinants of HF between men and women may be relevant for further therapeutic strategies including the inhibition of gal-3.

## 1. Introduction

Post-infarction left ventricular remodeling (LVR) is a complex process consisting of myocardial hypertrophy, fibrosis, ventricular dilatation and apoptosis at the cellular level. The remodeling process appears to be more favorable in women than in men, but it is known that men have a higher incidence of adverse outcomes after acute myocardial infarction (MI) [1]. It is still unclear whether sex differences can modulate the response to reperfusion treatment after primary percutaneous coronary intervention (PPCI) and outcome, but recent studies have shown that differences in mortality are still evident [1,2]. The response of the myocardium to ischemic injury and cardiovascular stress also differs between men and women. Women’s hearts tend to undergo concentric remodeling, while men’s hearts more often undergo eccentric remodeling. Heart failure (HF) is the most common clinical presentation after MI in women [3,4]. Sex-specific differences in the frequency distribution of candidate gene variants and their interactions in the process of LV remodeling probably lead to the pathological phenotype of HF [5]. In particular, HF with preserved left ventricular ejection fraction (HFpEF) or middle-range EF (HFmEF) affects women more frequently than men in a ratio of 2:1 [4]. The underlying mechanisms are still unclear, although gender-specific differences in cardiac structure, function and aging have been described [3,4]. Doppler echocardiography with advanced technology allows both accurate and reproducible determination of LV structure and function during a remodeling process and detection as well as the characterization of HF in early and late stages after the first MI [6,7].

Biological sex differences in HF biomarkers may result from differences in genetic makeup and the direct effects of sex hormones, and also indirectly from differences in fat distributions between man and women. Galectin-3 (gal-3), a β-galactoside-binding lectin, encoded by the *LGALS-3* gene, is involved in various inflammatory processes as one of the macrophage activators, in particular, in myocardial fibrosis, predicting and contributing to cardiac dysfunction and adverse cardiovascular outcomes in patients with coronary artery disease (CAD) [8]. There are several gene variants associated with circulating gal-3 levels that may contribute to the process of inflammation and fibrosis during LV remodeling after MI and the occurrence of HF [5]. Gal-3 was found to be significantly associated with incident HF only in women [4]. It has recently been shown that gal-3 levels are 2.1 times higher in women than in men [8,9]. However, given the clinical importance, the links between the regulation of gal-3 at both levels, mRNA level and plasma protein level on the one hand and LV function and the occurrence of HF in men and women on the other hand, are not clear enough.

We investigated sex differences in clinical, coronary angiographic, structural and functional characteristics, *LGALS-3* mRNA expression in peripheral blood mononuclear cells (PBMCs) and plasma gal-3 concentration during the remodeling process in patients after the first MI. We investigated the occurrence of HF during the six-month follow-up and the role of gal-3 in the development of HF.

## 2. Materials and Methods

### 2.1. Study Population

Between October 2011 and December 2013, 181 consecutive patients with first acute MI, ST-elevation myocardial infarction (STEMI) and non-ST-elevation myocardial infarction (NSTEMI) were prospectively studied by Doppler echocardiography 2–4 days after hospital admission and after 6 months of follow-up in the Coronary Care Unit in the Department of Cardiology, University Clinical Center “Zvezdara”, Belgrade, Serbia.

We conducted this study in compliance with the ethical standards of the Regional Bioethics Committee of University Clinical Hospital Center “Zvezdara”, Belgrade, Serbia, and the World Medical Association Declaration of Helsinki on Ethical Principles for Medical Research Involving Human Subjects. All subjects gave their informed consent to participate in the study and an information letter was given to them.

The study was approved by the Ethics Committee of the University Clinical Center “Zvezdara”, Belgrade, Serbia (approval code: NoIRB00003818; approved by Federalwide Assurance–FWA00006109; date of approval: 29 June 2011).

Inclusion criteria were chest pain lasting > 30 min, ST elevation > 0.2 mV in at least two contiguous leads in the initial electrocardiogram, elevated markers of myocardial necrosis and treatment with PPCI within 6 h. Symptoms of acute MI may be atypical in more than 40% of women who have nausea, fatigue, dyspnea or may be asymptomatic; in our study, we included all diagnostic criteria for acute MI [10]. Exclusion criteria for all patients were age over 70 years, history of MI or other cardiac disease (e.g., HF, cardiomyopathies, significant valvular heart disease, myocarditis) significant arrhythmias (including atrial fibrillation) or cardiogenic shock or previous implantation of a pacemaker or other device. Those exclusion criteria are very important for the study, considering that the goals of our research were to evaluate the structural and functional characteristics of the heart after the first MI. Those characteristic and hemodynamic parameters are already altered and variable in patients with previous or concomitant heart disease such as valvular, cardiomyopathy or rhythm disturbances [11,12]. Age >70 years compromises the LV diastolic function by altering the late diastolic (atrial) filling velocity and the deceleration time of early diastolic filling [11]. Patients with STEMI obtained coronary angiography and were treated with PPCI within 6 h from symptom onset, whereas patients with NSTEMI were treated promptly with optimal medical therapy with coronary angiography in severe clinical or electrical instability within the first 2 h or within the first 24 or 72 h, according to European Society of Cardiology and Heart Association guidelines [12]. Baseline characteristics of the patients were collected and the study population was divided into two groups: men and women.

### 2.2. Laboratory Analysis

All standard biochemical analyses were performed in the hospital laboratory following standard laboratory procedures, and included biomarkers for myocardial necrosis such as troponin (Tn), creatine kinase (CK) and creatine kinase MB (CKMB) on admission, the peak value during hospital treatment and the value on the day of discharge.

### 2.3. Echocardiography

The first acquisition of 2D echocardiography was performed within 2–4 days of admission. A repeat echocardiographic examination was performed after 6 months. Standard M-mode and 2D, color, pulse-wave and continuous-wave Doppler images were acquired and stored in cine-loop format, and data analysis was performed offline.

Echocardiographic data were obtained using a commercially available second harmonic imaging system, Toshiba XG/Artida, equipped with a 2.5/3.0 MHz probe (Toshiba Medical Systems, Otawara, Japan). All echocardiographic measurements were obtained according to the guidelines of the American Society of Echocardiography and the European Association of Cardiovascular Imaging [6]. Using M-mode images, LV end-diastolic (LVEDD) and end-systolic diameters (LVESD), as well as the diastolic wall thickness of the interventricular septum (IVS) and posterior wall (PW), were determined from the parasternal view in the long axis at the level of the papillary muscle. From the apical four- and two-chamber views, LV end-diastolic and end-systolic volumes (LVEDV and LVESV) were measured. LV ejection fraction (LVEF) was calculated from the apical 4- and 2-chamber views using the modified Simpson method (multiple slices). Both LV volumes were indexed by body surface area (BSA) (LVEDVI and LVESVI). LV mass index (LVMI) was calculated as previously described and relative wall thickness (RWT) was calculated using the formula (2 × PW/LVDD), where PW is posterior wall thickness at end-diastole [6].

The change (∆) in cardiac parameters during 6 months was calculated as the difference between the values at 6-month follow-up points and the baseline points.

Left atrial (LA) volume was measured by the modified Simpson’s method, using the largest diameter of the LA during its systole, as previously described, and LA volume was indexed by BSA (LAVI) [6,7].

Pulsed-wave Doppler of the mitral valve inflow was obtained by placing the Doppler sample volume between the tips of the mitral leaflets. Peak early (E) and late (A) diastolic velocities and the deceleration time (DT) were recorded from the transmitral spectral trace. Peak systolic velocity (s) and early and late diastolic annular velocity (e, a) were obtained from annular tissue Doppler imaging (TDI). The ratio E/e was calculated as the diastolic LV index reflecting end-diastolic LV pressure. The tricuspid regurgitation peak velocity was measured and pulmonary systolic pressure was calculated [7]. Moderate diastolic dysfunction was defined as an E/A ratio greater than 1 with a level of annular septal E/e greater than 13 [7,11].

Myocardial tissue deformation (strain) was calculated during systole by speckle tracking echocardiography using the Toshiba 2D Tissue Tracking System. Global longitudinal strain (GLS), as a measure of global longitudinal myocardial deformation as well as longitudinal LV function, was obtained from three conventional apical imaging planes (four-chamber, two-chamber and apical long-axis views) and the average value was calculated. Peak systolic strain was defined as the highest deformation during systole [12].

The diagnosis of HF was confirmed by physical examination and Doppler echocardiographic guidelines, according to the NYHA functional classification [13].

### 2.4. Coronary Angiography, Percutaneous Coronary Intervention and Medication

Coronary angiography was performed by standard trans-femoral or trans-radial arterial catheterization. All patients received an intravenous bolus injection of 3000 IU heparin before angiography. Quantitative coronary angiography (QCA) was performed using the Innova^TM^ IGS 520 Image Guided System (GE HealthCare Technologies, Inc., Chicago, IL, USA), which is calibrated based on coronary guidance [14]. Two observers marked the region with the most severe CAD. Significant coronary stenosis was defined as a narrowing of the luminal diameter of >70% on the angiogram. The TIMI (thrombolysis in myocardial infarction) grade was determined before and at the end of the PPCI procedure. Successful angioplasty was defined by the combination of post-procedural TIMI flow grade 3 and residual stenosis <30%. The status of culprit lesions and the number of stents and their positions were determined. Time to reperfusion was defined as the interval between the onset of symptoms and the first balloon inflation. Syntax scores were calculated considering the functional and anatomical components, including the presence of bifurcations, total occlusions, thrombi, calcifications and small vessels [15].

### 2.5. Reverse Transcripion–Quantitative Real-Time PCR (RT-qPCR)

Whole peripheral blood samples for total RNA extraction were collected from patients in the early phase of MI (within 2–4 days). The PBMCs were separated using Lymphocyte Separation Medium (PAA, GE Healthcare) and the total RNA extraction from the PBMCs was performed using TRIzol^®^ Reagent (Invitrogen, Thermo Fisher Scientific, Waltham, MA, USA) according to the manufacturer’s instructions within 30 min of sample collection. The recombinant Thermo Scientific™ RiboLock RNase Inhibitor effectively protects RNA from degradation at temperatures up to 55 °C (Thermo Fisher Scientific, Waltham, MA, USA). RNA samples were stored at −80 °C prior to use. The RNA quantification was evaluated using the BioSpec-nano spectrophotometer (Shimadzu Corporation, Kyoto, Japan). The integrity of the RNA was assessed by chip electrophoresis using the RNA 6000 Nano Kit on the 2100 Bioanalyzer system (Agilent Technologies, Inc., Santa Clara, CA, USA). All samples yielded total RNA of satisfactory quality with an RNA Integrity Number (RIN) value of 8–9 and were converted to cDNA.

One µg of RNA was treated with Thermo Scientific™ DNAse I and reverse transcription was performed using a first-strand cDNA synthesis kit with Thermo Scientific™ oligo(dT)_18_ primer and Thermo Scientific™ RevertAid Reverse Transcriptase (Thermo Fisher Scientific, Waltham, MA, USA) in a 20 µL reaction volume, in accordance with the manufacturer’s instructions. Real-time PCR was performed in duplicate on the ABI Real-time 7500 system (Applied Biosystems, Thermo Fisher Scientific, Waltham, MA, USA).

The detection of *LGALS-3* gene and the reference gene *Peptidylprolyl isomerase A* (*Cyclophilin A*)’s relative expressions was determined using the TaqMan^®^ gene expression assays Hs00173587_m1 and Hs99999904_m1, respectively (Applied Biosystems, Thermo Fisher Scientific, Waltham, MA, USA), as previously described [16].

### 2.6. Quantification of pGal-3 Levels

Whole peripheral blood samples, collected in baseline points (within 2–4 days), were centrifuged within 30 min of collection, and the plasma samples were stored at −80 °C prior to use. Gal-3 concentrations were determined using the Elabscience^®^ Human GAL3 (galectin-3) ELISA Kit (Beijing, China) according to the manufacturer’s instructions. Perkin Elmer Wallac 1420 Victor2 Microplate Reader (PerkinElmer, Inc., Waltham, MA, USA) was used to measure optical density at 450 nm. Using a four-parameter logistic curve-fit standard curve, gal-3 was determined using a number that represented the averaged duplicate readings of optical density measured for each sample (My Assays: online data analysis, as previously described [17]. Available at: https://www.myassays.com/four-parameter-logistic-curve.assay, accessed on 15 September 2022).

### 2.7. Statistical Analysis

All categorical variables are expressed as percentages and all continuous variables as mean ± standard deviation (SD) or median (25–75th percentile). Comparisons of categorical variables were estimated using Pearson’s Chi-squared (χ^2^) test. Means of normally distributed continuous variables were compared using the unpaired *t*-test. Medians of skewed continuous variables were compared using the non-parametric Mann–Whitney U test. *p* values < 0.05 were considered statistically significant.

Spearman’s rank correlation coefficients (R) and linear regression analyses were performed to investigate risk factors for HF occurrence after 6 months, while adjusting for other variables by using the enter method. Statistical analysis was performed with the use of the SPSS software package for Windows version 20.0 (SPSS Inc., Chicago, IL, USA).

The statistical power of the study for the observed associations was calculated using the post hoc power clinical calculator (available at https://clincalc.com/stats/Power.aspx, accessed on 25 August 2024).

## 3. Results

### 3.1. Sex Differences Between Clinical, Doppler Echocardiographic and Angiographic Data

Of the 181 patients enrolled in the study, 44 were female (25.74%) and 137 were male (74.27%). Baseline demographic, clinical and laboratory parameters are shown in Table 1. Women were older, had higher total cholesterol and LDL cholesterol and lower hematocrit. Coronary angiographic indices during primary percutaneous interventions and post-MI medication are shown in Table 2. There were no significant differences between men and women in the following: time from “door to balloon”, percentage of patients with anterior wall infarcts and percentage of successful reperfusion. In the group of patients with single-vessel and multi-vessel coronary disease, similar numbers of women and men were represented. There were no gender differences in the severity of CAD according to the syntax score or in the reperfusion pattern, represented as TIMI flow according to PPCI.

Medical treatment in hospital was similar in men and women, including β-blockers, ACE inhibitors, antiplatelet agents and diuretics.

Echocardiographic Doppler variables for both sexes in the study populations are shown in Table 3. Baseline and follow-up values and the difference (∆) in volumes and function during the six-month follow-up are also shown. LVEDVi and LVESVi were significantly lower in women than in men, both in the acute phase of MI (58.2 ± 13.1 vs. 46.3 ± 11.1, *p* < 0.001; 33.7 ± 9.5 vs. 27.0 ± 9.2, *p* = 0.001, respectively) and at follow-up (60.2 ± 15.3 vs. 49.2 ± 16.2, *p* < 0.001; 35.3 ± 13.7 vs. 28.1 ± 13.6, *p* = 0.004, respectively). At follow-up, a similar slight increase in LVEDVi and LVESVi was observed in both sexes; however, the results were not statistically significant.

The changes in volume indices as well as longitudinal deformation (GLS) showed a similar degree of LV remodeling. LVEF was equal in men and women in the acute phase, but a greater increase was observed in women over six months, although the results did not reach statistical significance. The distribution of patients with criteria for moderate to severe LV diastolic dysfunction (DD) differed significantly between men and women at 6 months.

### 3.2. Sex Differences Between Cardiac Biomarkers in Study Patients

**Figure 1:**
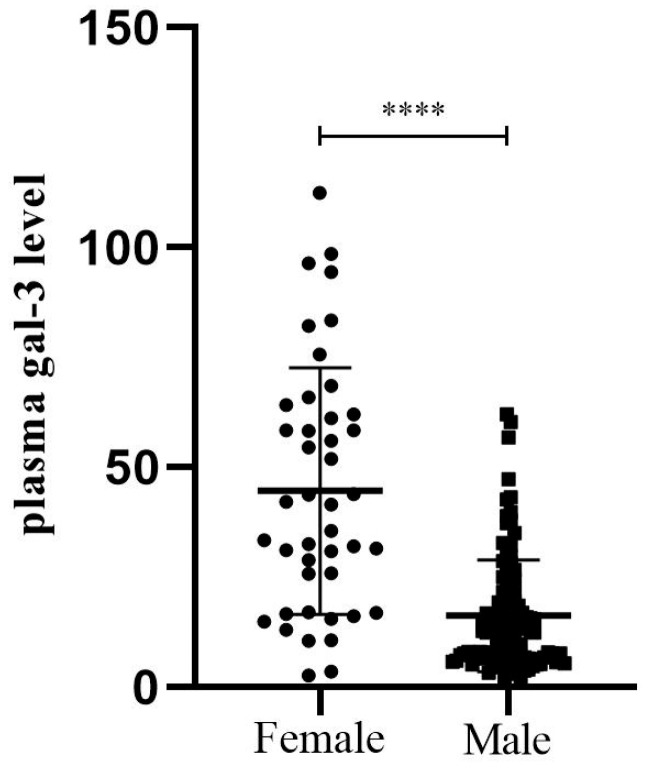
Plasma gal-3 levels in patients six months post-MI according to gender. Female patients had significantly higher plasma gal-3 levels compared to male patients (44.66 ± 28.04 ng/mL vs. 16.30 ± 12.68 ng/mL, respectively, *p* < 0.0001). **** *p* ≤ 0.0001.

We compared the differences between men and women in the levels of biomarkers of myocardial lesion and necrosis, including high-sensitivity troponin (HST), CKMB, C-reactive protein (CRP) and additional markers of renal function (electrolytes, urea, creatinine, glomerular function). The only biomarker that differed significantly between the sexes was gal-3, with a higher plasma concentration in women (44.66 ± 28.04 ng/mL vs. 16.30 ± 12.68 ng/mL, *p* < 0.0001; Figure 1). We had a study power of >80% for the observed association at a significance level of *p* < 0.05.

The level of gal-3 correlated with urea and creatinine levels (*p* < 0.001 and *p* = 0.045, respectively). The level of gal-3 correlated with urea and creatinine level (*p* < 0.001 and *p* = 0.045, respectively); significant differences were found in gal-3 level when it comes to renal function calculated according to glomerular filtration rate (GFR) (32.99 vs. 22.29, *p* = 0.025). A significant correlation was found between the level of HST at discharge from the hospital and gal-3 plasma concentration in the whole study group (*p* = 0.015), with no significant difference between men and women.

**Figure 2:**
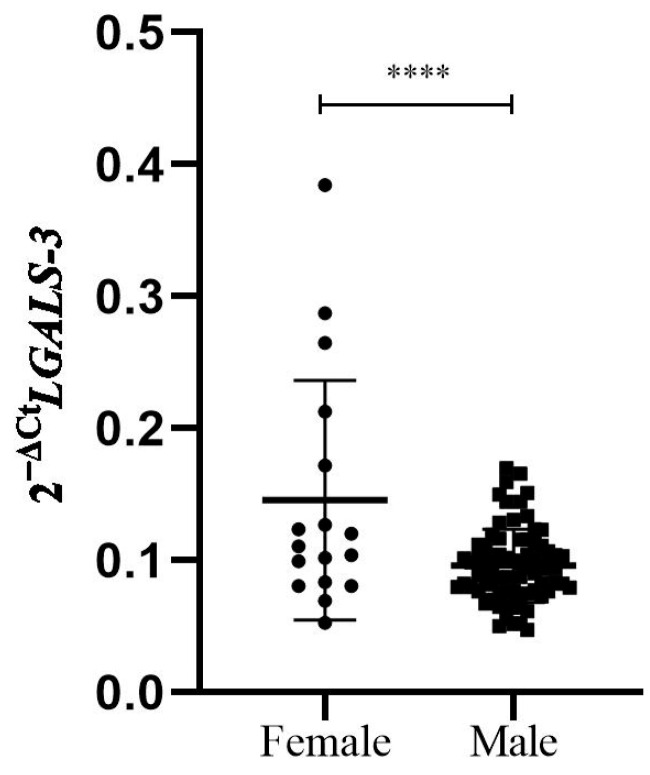
Relative *LGALS-3* mRNA expression in PBMCs of MI patients, according to gender. The cDNAs from human PBMCs (n = 92) were used to quantify gene expression. The relative expression of mRNAs between groups was calculated using a Mann–Whitney *U* test and presented in comparison to *Cyclophilin A* mRNA. The delta Ct value was calculated from the difference between the Ct value of the gene of interest and that of the reference gene, *Cyclophilin A*. Data are presented as 2^−∆Ct^, with means for both groups (females—circles; males—squares) with 95% CI. Significant upregulation of *LGALS-3* mRNA was detected in PBMCs from female patients (n = 17) compared to male patients (N = 75) (0.1456 ± 0.0907 vs. 0.0959 ± 0.0276, respectively, *p* = 0.0001). **** *p* ≤ 0.0001.

The relative expression levels of *LGALS-3* mRNA in PBMCs from 92 patients 6 months after the first MI were significantly higher in women than in men (0.1456 ± 0.0907 vs. 0.0959 ± 0.0276, *p* = 0.0001) (Figure 2). We had a study power of 63% for the observed association at a significance level of *p* < 0.05.

### 3.3. Correlations Between Gal-3 Levels and Parameters of LV Remodeling and Function, with Sex Differences

**Figure 3:**
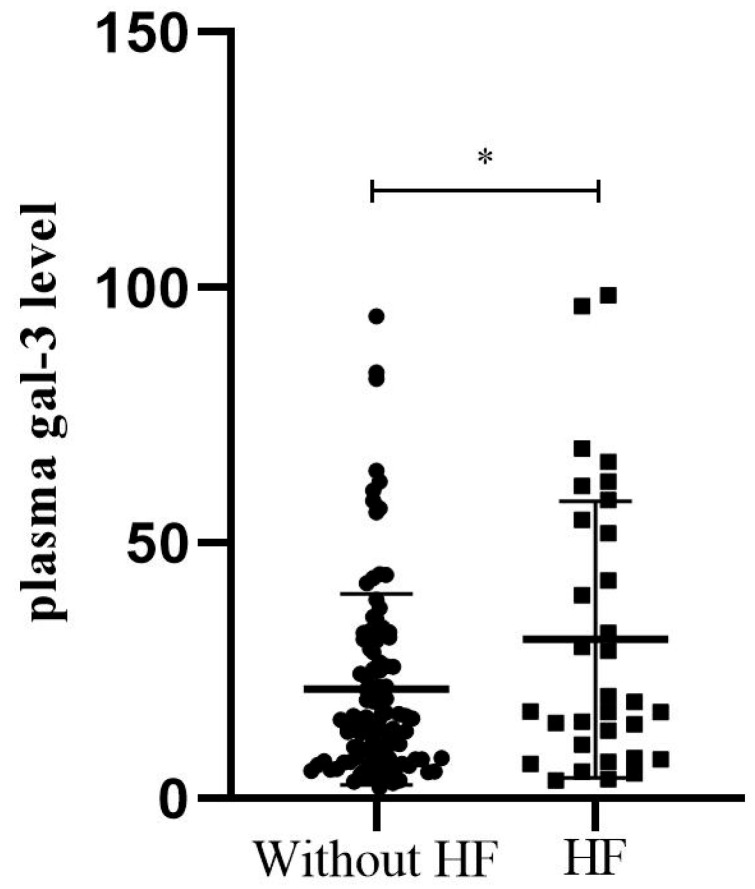
Plasma gal-3 levels in patients six months post-MI according to the heart failure occurrence six months after the first MI. Patients with heart failure six months post-MI had significantly higher plasma gal-3 levels compared to patients without heart failure (31.14 ± 27.09 ng/mL vs. 21.39 ± 18.71 ng/mL, respectively, *p* = 0.024). * *p* < 0.05.

**Figure 4:**
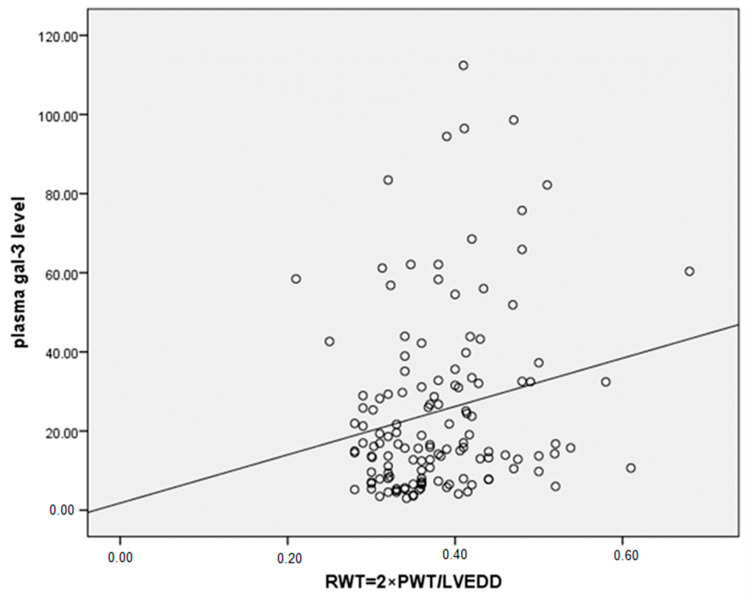
Pearson two-tailed correlation between plasma gal-3 levels and relative wall thickness (RWT) as marker of LV hypertrophy in patients six months after first MI (r = 0.225, *p* = 0.02).

**Figure 5:**
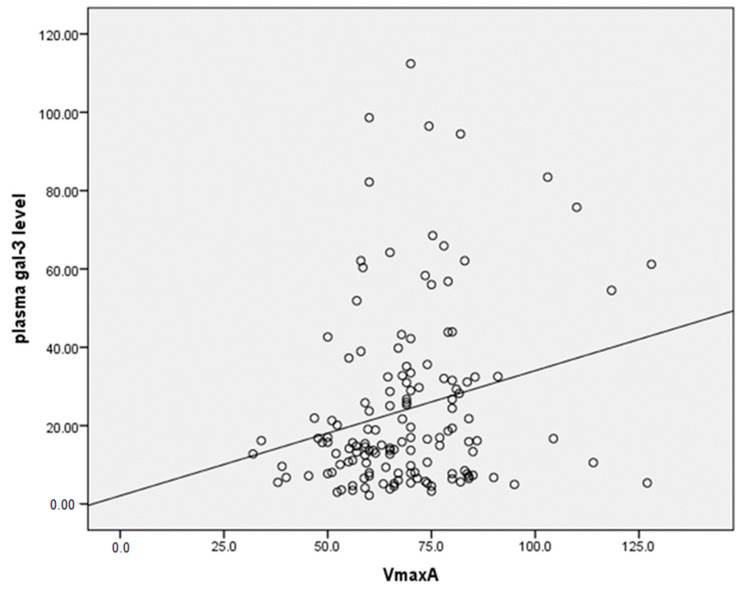
Pearson two-tailed correlation between plasma gal-3 level and maximal velocity of late diastolic LV filling (VmaxA), (r = 0.231, *p* < 0.006).

The number of patients with HF 6 months after MI was significantly higher in the female group than in the male group (37.5% vs. 19.5%, *p* = 0.02). We found higher plasma gal-3 levels in patients with HF compared to patients without HF six months after MI (31.14 ± 27.09 vs. 21.39 ± 18.71, *p* = 0.024) (Figure 3). The incidence of LV hypertrophy (LVH) was also significantly higher in women compared to men (70.0% vs. 44.6%, *p* = 0.03). We found significant differences between men and women in the parameters of LV diastolic dysfunction, such as significantly higher late mitral inflow velocity (67.09 vs. 74.14, *p* = 0.02), E/e ratio (10.1 vs. 11.54, *p* = 0.015) and pulmonary systolic pressure (34.2 vs. 37.53, *p* = 0.024). RWT, an index of concentric LV remodeling, was found to correlate significantly with plasma gal-3 levels in patients after the first MI (r = 0.225, *p* = 0.02) (Figure 4). A significant correlation was found between plasma gal-3 levels and the maximal velocity of late diastolic LV filling (r = 0.231, *p* < 0.006) (Figure 5).

## 4. Discussion

Galectin-3, a regulatory protein, is elevated in both acute and chronic heart failure and is involved in the post-injury inflammatory pathway leading to myocardial tissue remodeling [8]. Gal-3 plays an important role in the pathogenesis of fibrotic heart disease as a factor that promotes the development of inflammation and worsens prognosis [18]. In our study group, significantly higher levels of gal-3 were found in women compared to men, in patients who developed HF 6 months after MI. Higher levels of gal-3 were found in patients with LV hypertrophy and diastolic LV dysfunction, the predominant pattern of LV remodeling in women leading to HF. Therefore, gal-3 could potentially be a biomarker for HF, especially in female patients after the first MI.

It is already known that gender has a profound effect on remodeling in aging hearts free of any cardiovascular disease [19]. The cardio-protective role of estrogen, the main circulating female sex hormone, is the most investigated pathophysiological mechanism intended to explain the gender-related differences in the remodeling process [19]. High vascular stiffness, which leads to diastolic dysfunction, elevated blood pressure, and the development of HFpEF, is a symptom of estrogen deficiency [19,20]. Furthermore, estrogen is known to modulate natriuretic peptides and activate angiogenesis, both of which support the hypertrophic heart’s increased oxygen demand [19,20,21]. Because the women in our study were older, they had a deficiency in estrogen. Mallat et al. found that the apoptotic index was three-fold higher in aging men than in women [21]. Post-mortem data suggest that men and women modulate the apoptotic signaling pathway differently in the peri-infarct region. Women appear to be at least partially protected from the ischemia-induced apoptotic cascade [20]. Gender-specific differences in cardiac remodeling may explain the differences in clinical outcome after MI [22]. However, although the remodeling process appears to be more favorable in women than in men, it is known that women are more frequently affected by adverse outcomes after myocardial infarction [23,24]. Data from the PARADIGM-HF and ATMOSPHERE studies have shown that women with HF have more severe symptoms and signs, higher health-related quality of life and greater functional impairment than men, despite better survival and fewer hospitalizations than men [25,26]. Once the acute tissue damage has passed, adaptive remodeling is responsible for maintaining myocardial morphology and function. During further maladaptive remodeling, ventricles tend to dilate while the lost cardiomyocytes are gradually replaced by cardiac fibroblasts and collagen. More common patterns of remodeling in women are not dilatation of the heart chambers, but hypertrophy of the myocardium surrounding the infarct and functional heart failure [27]. Experimental studies suggest a relatively analogous pattern of global remodeling, but there are sex-specific differences in the remaining myocardial tissue alterations such as the size of cardiac myocytes or interstitial collagen content [27,28]. In our study, the increased incidence of HF in women six months after the first MI may be related to greater cardiac hypertrophy in women and increased plasma gal-3 concentration. Similar to our results, Grandin et al. showed a significant graded relationship between the level of plasma gal-3 concentration within the first week after MI and the development of HF. In a case–control study, PROVE IT-TIMI22, the authors concluded that gal-3 as a biomarker of adverse LVR was associated with the risk of developing HF after acute coronary syndrome [29].

Myocardial injury is probably the trigger for the increased release of gal-3 from macrophages by different mechanisms [30]. In our study, the positive correlation between HST concentration as a biomarker of myocardial necrosis and the plasma concentration of gal-3 contributes to this claim.

To improve our understanding of sex-specific mechanisms related to HF risk, recent studies have examined sex-specific associations of circulating biomarkers. Much of the evidence showed that the major HF-related pathophysiological mechanisms sensed by biomarkers are broadly similar in men and women, but the profibrotic marker gal-3 is more strongly associated with HF risk in women than in men [31]. Encoded by a single gene, *LGALS-3*, galectin-3 is upregulated in injured and remodeled tissue and serves as a marker of fibrosis and inflammation [31]. In our study, the relative expression levels of *LGALS-3* mRNA were significantly higher in women, as was the level of circulating gal-3. The presence of LV hypertrophy is more common in women, so given the already well-documented effects of gal-3, it may play a more important role in the development of HF, in which inflammation and fibrosis are the dominant mechanisms [27,31,32].

Future studies will clarify whether fibrotic mechanisms play a greater role in the pathophysiology of HF in women. In the absence of estrogen, fibrotic processes such as vascular stiffness, increased blood pressure and LVDD contribute to HF [32]. The mean age of women in our study was 57.8 ± 7.6 years, i.e., the majority of women were postmenopausal, without estrogen protection. Considering the lower rates of structural loss with the preservation of cardiac dimensions and volumes, the reason for the higher incidence of HF in women was possibly unchanged or worsened LVDD during the follow-up period. The E/e index, which reflects LV filling pressure, was significantly higher in women compared to men 6 months after MI [33,34]. The present study showed a very close correlation between the level of circulating gal-3 and the grade of LV diastolic dysfunction in all study groups. Similar results were presented in the recent study conducted by Ansari et al., in which the authors suggested that gal-3 is a promising biomarker that has the potential to classify patients with HFpEF syndrome and is very helpful in cases where the interpretation of echocardiography is limited [34]. Increasing values of serum gal-3 may reflect the progressive course of HFpEF, as classified by echocardiographic Doppler grades of diastolic dysfunction [33,34]. In the study patients, close correlations were found between echocardiographic parameters of LV diastolic function such as the maximal velocity of early (VmaxE) and late diastolic filling (VmaxA), the E/e ratio on the one hand, and the plasma concentration of gal-3 on the other. Moreover, the level of plasma gal-3 concentration was associated with the degree of LVDD, similar to our results [34,35]. One of the speculations is that gal-3 may lead to a downregulation of the sarcoplasmic reticulum Ca-ATPase (SERCA) and thus to diastolic dysfunction by increasing the inflammatory process and fibrosis [36].

There is ample evidence that myocardial salvage after the first acute MI is more favorable in women, especially due to the better reperfusion pattern after PPCI [32,37]. In our study, coronary angiographic characteristics, including the number of impaired vessels, as well as lesion severity and success of the PCI procedure, were similar in men and women.

Over the past decade, studies have documented gender differences in the administration of reperfusion therapy for patients with acute MI, suggesting that women are more likely to experience treatment delays and are less likely to receive aspirin and β-blockers [32,38]. In the present study, treatment strategies in the acute setting were standardized and therefore comparable between the two genders. In addition, there were no significant differences in the medication during hospitalization.

There are some limitations of the present study. Even though the study is prospective, the number of patients, especially females, is limited. The ratio between the number of men and women in the study group was disproportionate, especially for the study of sex-related differences. We do not have detailed information on patients’ use of the recommended medications before enrollment in the study and during the six-month follow-up. In our study, blood samples were collected only once, six months after the first myocardial infarction, and *LGALS-3* mRNA expression and gal-3 levels in plasma were determined from those samples. Therefore, we were unable to determine the dynamic of *LGALS-3* mRNA expression over time, that is, to assess the trend from the early to late phase of myocardial infarction. Replication and validation in a larger sample is needed to more accurately estimate the strength of the associations.

## 5. Conclusions

Structural changes in LV in the acute phase and at 6 months after acute myocardial infarction are less pronounced in women than in men, but the degree of LV remodeling was similar. LV hypertrophy and LVDD patterns were more common in women than in men. After a follow-up period of 6 months, the development of HF was significantly more frequent in women than in men. *LGALS-3* mRNA expression was significantly higher in PBMCs from women, as was the plasma concentration of gal-3. The plasma concentration of gal-3 was significantly higher in patients who developed HF at 6 months. Future studies should investigate the clinical value of gal-3, its different dynamics in men and women, its importance in the development of HF and the prognosis of patients after the first MI. Further investigations should be conducted to confirm galectin-3 as a marker or potential therapeutic target.

## Figures and Tables

**Table 1:** Demographic characteristics, risk factors and laboratory findings.

Variable	Gender		*p* Value
	Male (*n* = 137)	Female (*n* = 44)	
Age (years)	54.8 ± 8.2	57.8 ± 7.6	0.034
Hypertension, *n* (%)	78 (57.8)	25 (58.1)	0.967
Cigarette smoking, *n* (%)	82 (62.6)	25 (62.5)	0.991
Diabetes mellitus, *n* (%)	48 (34.8)	14 (31.8)	0.718
Family history, *n* (%)	80 (62.5)	27 (67.5)	0.566
BMI (kg/m^2^)	27.8 ± 3.6	26.7 ± 4.4	0.125
NSTEMI, *n* (%)	28 (20.7)	7 (15.9)	0.483
Advanced Killip class, *n* (%)	15 (11.6)	4 (9.5)	1.000
Systolic blood pressure (mmHg)	129.9 ± 25.2	129.8 ± 23.8	0.988
Diastolic blood pressure (mmHg)	80.7 ± 16.1	79.7 ± 12.9	0.705
Total cholesterol (mmol/L)	5.42 ± 0.99	6.09 ± 1.37	0.004
LDL cholesterol (mmol/L)	3.54 ± 0.93	4.03 ± 1.27	0.027
Triglycerides (mmol/L)	1.93 ± 1.15	1.79 ± 1.02	0.546
Glucose (mmol/L)	8.63 ± 4.23	8.56 ± 4.54	0.646
CRP (mg/L)	10.1 (3.9–26.3)	8.1 (3.6–30.2)	0.991
Troponine (ng/mL)	149.00	114.80	0.728
Hematocrit (mL/L)	42.9 ± 3.7	39.6 ± 3.9	<0.001

Unpaired *t*-test was used to compare the values of continuous variables with a skewed distribution between males and females. Pearson’s Chi-squared (χ^2^) test was used for comparison of the categorical variables. Mann–Whitney *U* test was used to compare the values of normally distributed continuous variables between males and females. Results are presented as percentage (%), mean ± standard deviation (SD) or median (25–75th percentile); BMI—body mass index, NSTEMI—non-ST elevation myocardial infarction, LDL cholesterol—low density lipoprotein cholesterol, CRP—C-reactive protein; advanced Killip class is defined as Killip class ≥ 2.

**Table 2:** Angiography and therapy (medications).

Variable	Gender		*p* Value
	Male	Female	
Infarct/related artery			
No sign lesion, *n* (%)	2 (1.5)	0	0.958
LAD and LMCA, *n* (%)	46 (34.8)	17 (38.6)	
Lcx, *n* (%)	29 (22.0)	9 (20.5)	
RCA, *n* (%)	55 (41.7)	18 (40.9)	
Number of CAD			
Normal, *n* (%)	1 (0.7)	0	0.593
One vessel, *n* (%)	66 (49.3)	25 (56.8)	
Two vessel, *n* (%)	47 (35.1)	12 (27.3)	
Three vessel, *n* (%)	20 (14.9)	7 (15.9)	
Only infarct related, *n* (%)	65 (49.6)	23 (53.5)	0.660
TIMI after PPCI (0–1), *n* (%)	12 (10.6)	4 (9.8)	1.000
PPCI of non infarction, *n* (%)	5 (3.8)	1 (2.4)	1.000
Syntax score culpit	8 (5–14)	8 (5–10.5)	0.638
Syntax score total	13 (7–22)	13 (8–20)	0.973
Aspirin, *n* (%)	135 (99.3)	43 (100)	1.000
Clopidogrel, *n* (%)	134 (98.5)	43 (100)	1.000
LMWH, *n* (%)	131 (96.3)	42 (97.7)	1.000
ACE-I or ARBs, *n* (%)	131 (96.3)	38 (88.4)	0.062
β-blockers, *n* (%)	108 (80.0)	38 (88.4)	0.260
Diuretic, *n* (%)	25 (18.4)	6 (14.0)	0.504
Statins, *n* (%)	134 (98.5)	43 (100)	1.000

Results are presented as percentage (%) for categorical variables and median (25–75th percentile) for continuous variables; Pearson’s Chi-squared (χ^2^) test was used for comparison of the categorical variables and Mann–Whitney *U* test was used to compare the values of normally distributed continuous variables between males and females. LAD—left anterior descending artery, LMCA—left main coronary artery, Lcx—left circumflex artery, RCA—right coronary artery, PPCI—primary percutaneous coronary intervention, LMWH—low molecular weight heparin, ACE-I—angiotensin-converting enzyme inhibitor and ARBs—angiotensin II receptor blockers.

**Table 3:** Doppler echocardiography and spackle tracking: structural, functional characteristics.

Variable	Gender		*p* Value
	Male	Female	
LVEDVi (mL/m^2^)			
First week	58.2 ± 13.1	46.3 ± 11.1	<0.001
6 months	60.2 ± 15.3	49.2 ± 16.2	<0.001
Δ	1.48 ± 12.4	2.62 ± 12.4	0.610
LVESVi (mL/m^2^)			
First week	33.7 ± 9.5	27.0 ± 9.2	<0.001
6 months	35.3 ± 13.7	28.1 ± 13.6	0.004
Δ	0.83 ± 8.82	0.94 ± 9.26	0.944
LVEF (%)			
First week	44.2 ± 6.6	44.8 ± 8.5	0.591
6 months	45.6 ± 8.0 *	46.8 ± 10.3 *	0.492
Δ	1.26 ± 6.96	2.33 ± 7.18	0.395
SV (mL)			
First week	75.9 ± 17.1	66.5 ± 17.4	0.004
6 months	80.2 ± 17.6 *	71.6 ± 14.3	0.006
Δ	4.62 ± 19.21	3.19 ± 15.66	0.692
VmaxA (cm/s)			
First week	66.48 ± 14.96	77.18 ± 15.96 *	<0.001
6 months	67.14 ± 15.99	74.34 ± 19.37 *	0.015
Δ	0.66 ± 0.059	−2.84 ± 0.071	0.316
E/e (ratio)			
First week	9.87 ± 3.06	10.78 ± 3.10	0.104
6 months	10.16 ± 3.04	11.54 ± 3.53	0.015 *
Δ	0.489 ± 2.520	0.344 ± 3.416	0.777
SPVD (mmHg)			
First week	33.4 ± 6.7	35.7 ± 8.4	0.099
6 months	34.2 ± 7.2	37.6 ± 10.3	0.024*
Δ	1.03 ± 8.08	2.09 ± 11.78	0.567
GLS (%)			
First week	−8.41 ± 2.97	−9.34 ± 3.88	0.164
6 months	−9.59 ± 3.08 *	−9.76 ± 3.13	0.769
Δ	−0.91 ± 2.97	−0.38 ± 4.06	0.377
GRS (%)			
First week	17.75 ± 7.50	20.62 ± 8.88	0.052
6 months	20.75 ± 8.26 *	21.67 ± 8.81	0.551
Δ	3.00 ± 7.93	0.87 ± 8.80	0.181
DD, *n* (%)			
First week	7 (5.11)	3 (6.81)	0.48
6 months	8 (11.68)	8 (18.18)	0.014

*t*-tests were used for all between-group comparisons. LVEDVi—left ventricular end-diastolic left ventricular volume index, LVESVi—left ventricular end-systolic volume index, LVEF—left ventricular ejection fraction, E/e—ratio between early mitral inflow velocity and annular velocity, SV—stroke volume, VmaxA—maximal velocity of late diastolic filling, SPVD—right ventricle systolic pressure, GLS—global LV longitudinal strain, GRS—global LV radial strain, DD—diastolic dysfunction and Δ—chang es in cardiac parameters over a six month period, calculated as a difference between the value at six months points and baseline points. * *p* values < 0.05 were considered statistically significant.

## Data Availability

The original contributions presented in the study are included in the article, and further inquiries can be directed to the corresponding author.
